# Supplementary material for: The MTL200: a surface-based, probabilistic atlas of the medial temporal lobe
Source: bioRxiv. 2025 Dec 9:2025.12.04.692420. Preprint. [Version 1] doi: 10.64898/2025.12.04.692420 (PMC12707270; doi:10.64898/2025.12.04.692420)
Supplement: Supplement 1 [file media-1.pdf]

Supplementary Information

Supplementary Information for Faul et al. “The MTL200: a surface-based, probabilistic atlas of the medial temporal lobe.”

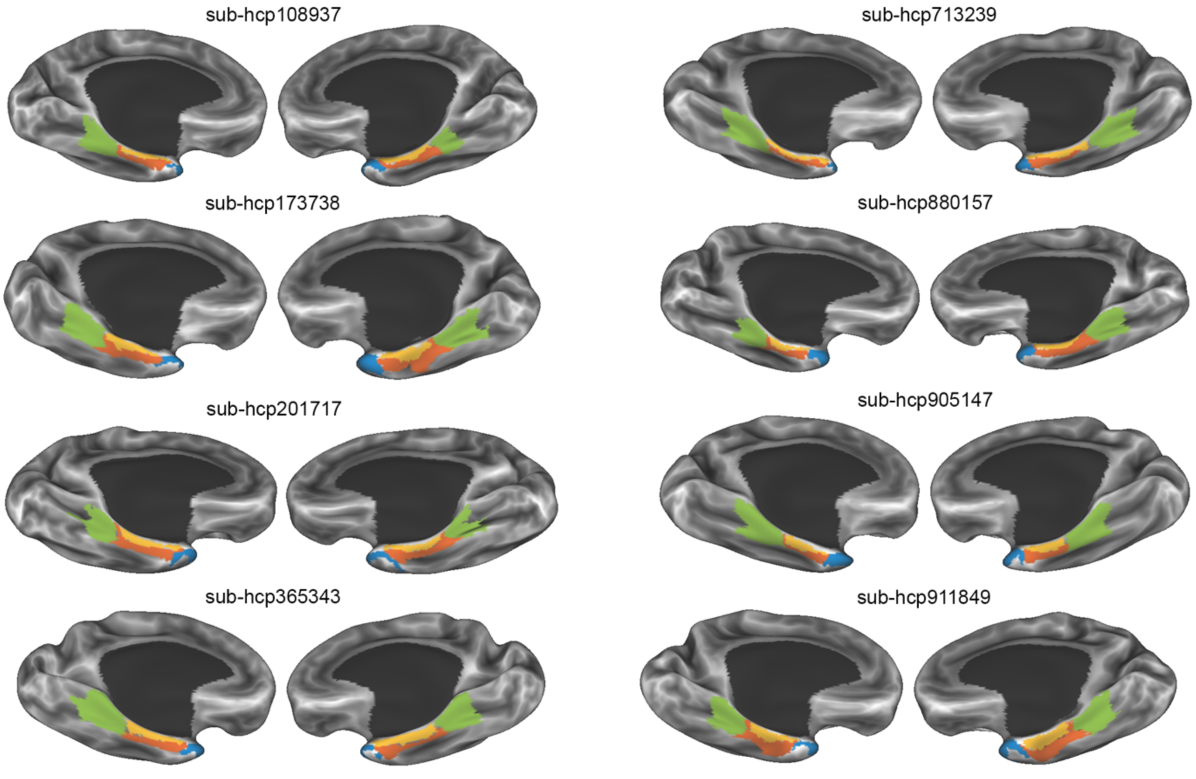

Fig S1. Parcellations on the cortical surface in eight example participants (medial view).

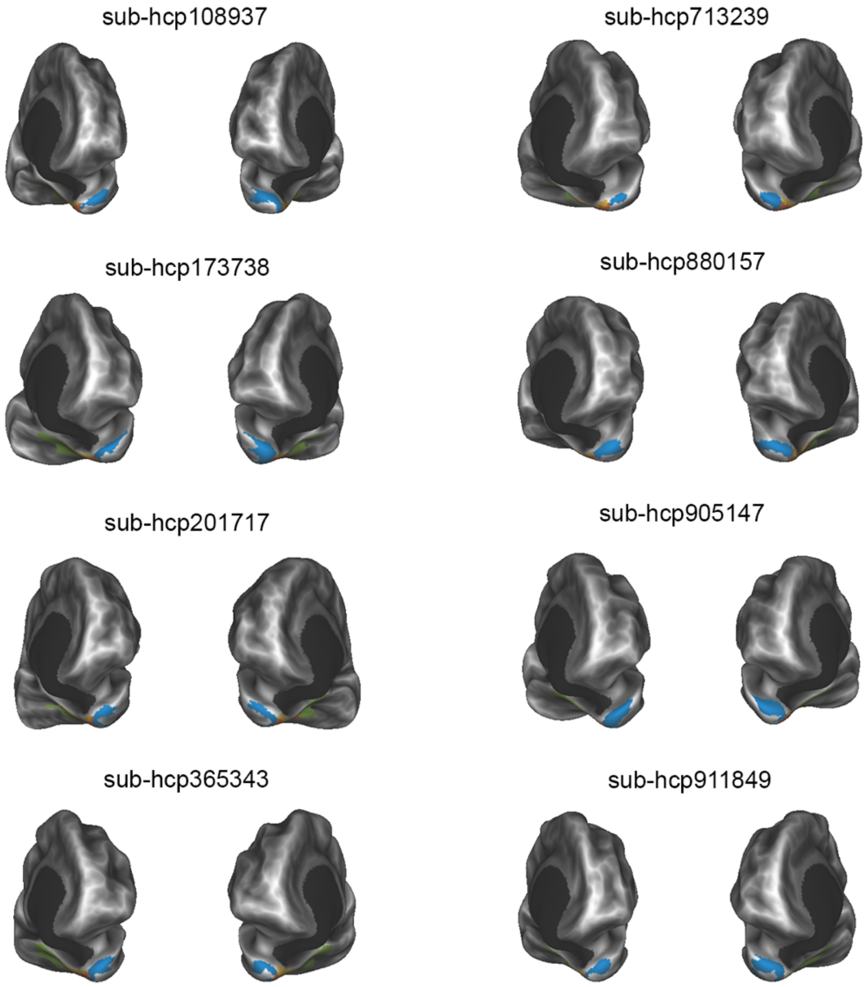

Fig S2. Parcellations on the cortical surface in eight example participants (frontal view).

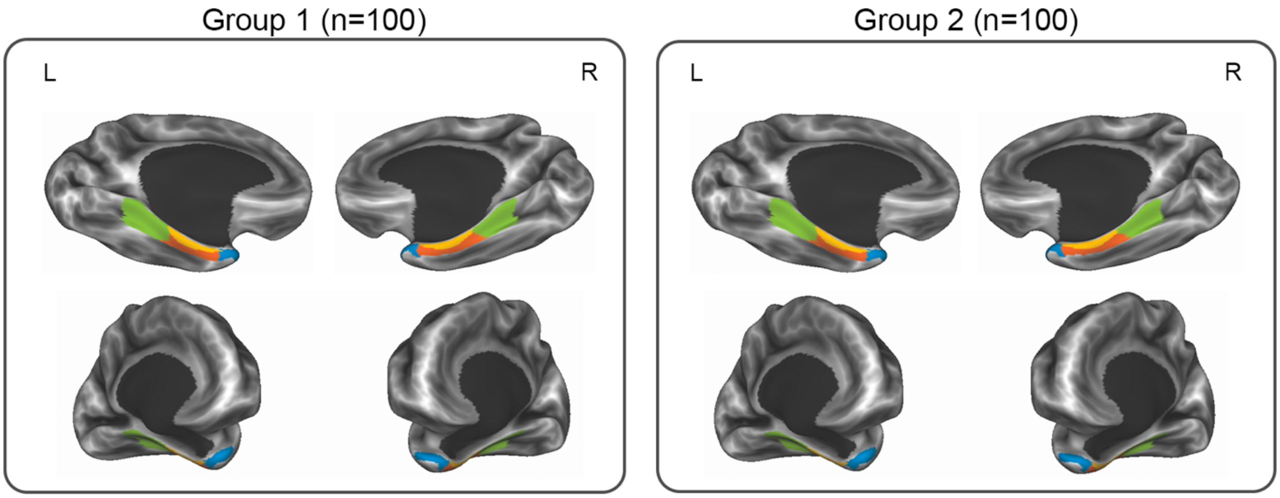

Fig S3. Split-half analysis demonstrates high reliability in the discrete segmentation generated across two partitions of participants.

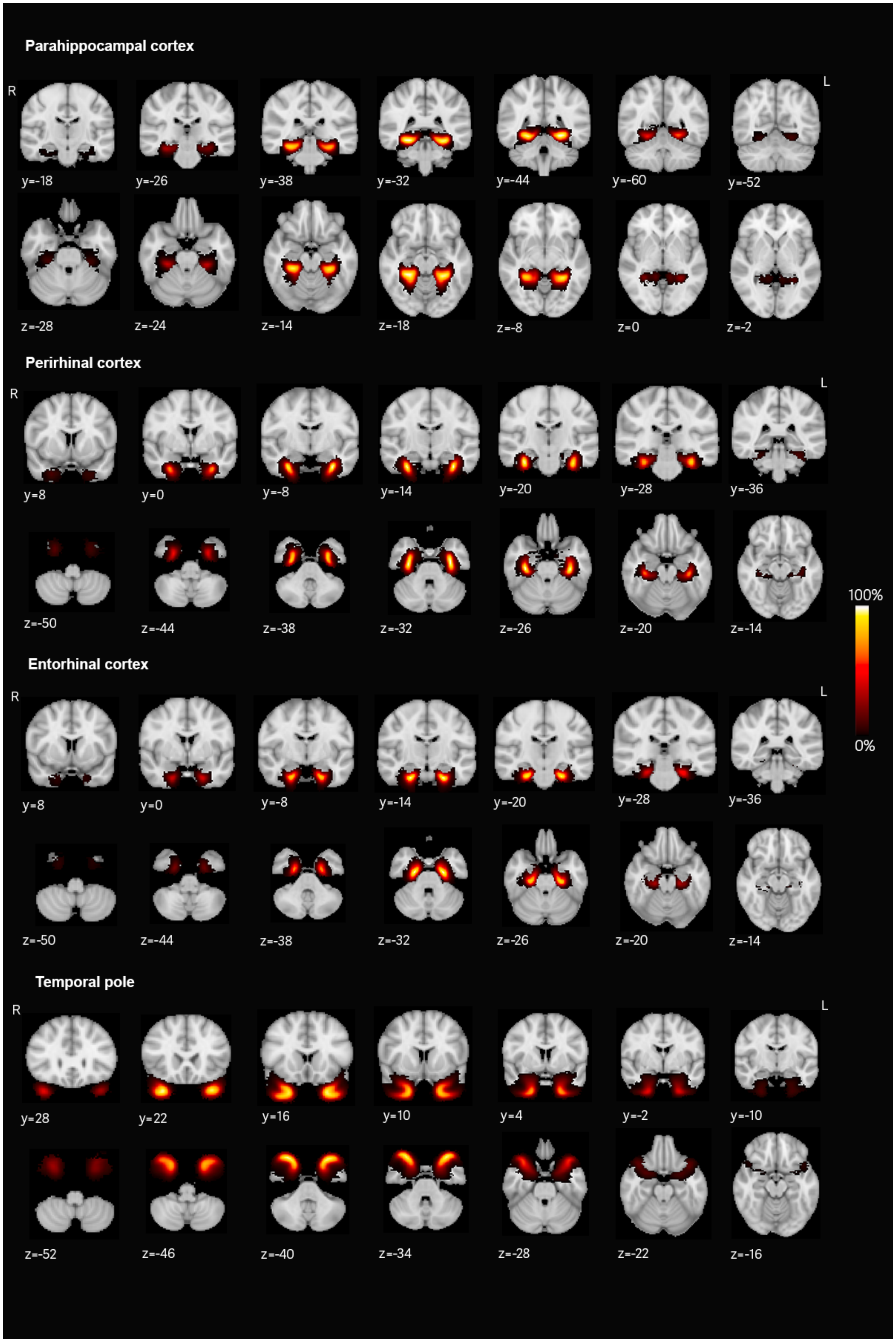

Parahippocampal cortex
R
L
y=-18
y=-26
y=-38
y=-32
y=-44
y=-60
y=-52
z=-28
z=-24
z=-14
z=-18
z=-8
z=0
z=-2
Perirhinal cortex
R
L
y=8
y=0
y=-8
y=-14
y=-20
y=-28
y=-36
z=-50
z=-44
z=-38
z=-32
z=-26
z=-20
z=-14
100%
0%
Entorhinal cortex
R
L
y=8
y=0
y=-8
y=-14
y=-20
y=-28
y=-36
z=-50
z=-44
z=-38
z=-32
z=-26
z=-20
z=-14
Temporal pole
R
L
y=28
y=22
y=16
y=10
y=4
y=-2
y=-10
z=-52
z=-46
z=-40
z=-34
z=-28
z=-22
z=-16

Fig S4. Volumetric implementation of the probabilistic atlas in MNI space.
